# An emotion regulation skills training for adolescents and parents: perceptions and acceptability of methodological aspects

**DOI:** 10.3389/fpsyt.2024.1448529

**Published:** 2024-12-10

**Authors:** Kristina Holmqvist Larsson, Maria Zetterqvist

**Affiliations:** ^1^ Department of Child and Adolescent Psychiatry in Linköping, Region Östergöland, Linköping, Sweden; ^2^ Department of Biomedical and Clinical Sciences, Center for Social and Affective Neuroscience, Linköping University, Linköping, Sweden

**Keywords:** adolescents, skills training, emotion regulation, child and adolescent psychiatry, group treatment

## Abstract

**Introduction:**

Difficulties with emotion regulation are associated with the development and maintenance of psychiatric symptoms. Focusing on emotion regulation can be beneficial when treating symptoms and behavioral problems. Here we describe a seven-session transdiagnostic approach to regulating emotions, delivered jointly to adolescents and parents in a child and adolescent psychiatric outpatient setting, and we explore the perceptions and acceptability of the methodological aspects of the intervention.

**Methods:**

Quantitative and qualitative data were analyzed. Participants (*n* = 117) were adolescents and parents.

**Results:**

Participants reported positive attitudes toward the skills training and would recommend it to others. Three overarching themes were developed. “Treatment components” relates to the content of the skills training. Participants appreciated focusing on ER skills but had different perceptions of the content. “The learning climate” contains process-related experiences, such as the skills trainers’ approach and the timing of the intervention. Participants preferred when skills trainers used self-disclosure. “Pedagogical aspects”, describes the structure of the training with perceptions of group constellations, length of sessions and homework. Adolescents particularly requested variety in the pedagogy and found long sessions to be challenging.

**Discussion:**

Recommendations for therapeutic practices are discussed.

## Introduction

Emotion regulation (ER) seems to play an important role in the development and maintenance of psychiatric symptoms (1, 2), and strategies for regulating emotions can be effective in treating symptoms and behavioral problems. Although there is not one generally agreed upon definition of emotion regulation, most definitions emphasize that emotion regulation is a mechanism that influences the intensity, duration, and expression of emotions (3). To regulate emotions, we use strategies (in relationship to a specific goal) to influence our emotions (4). In Gratz and Roemer’s (5) definition of adaptive emotion regulation, awareness and acceptance of emotions, and the ability to modulate emotional responses are emphasized, together with controlling impulsive behaviors to act according to a long-term goal or value, despite the presence of negative emotions.

Treatments that emphasize ER as a core component have successfully reduced addiction, self-harm, symptoms of posttraumatic stress disorder, symptoms of borderline personality disorder (BPD), and depression and anxiety, for example (6–10). Dialectical behavior therapy [DBT (11)] is one of the most disseminated treatments focusing on reducing ER difficulties. DBT has also shown promising results as a transdiagnostic treatment for depression and anxiety (12). Rathus and Miller (13) developed a specific manual for adolescents, DBT-A, which in randomized controlled trials has been shown to be superior to control conditions (enhanced care and supportive therapy) in reducing self-harm, for example (8, 14, 15). DBT is a comprehensive treatment consisting of individual therapy, a skills training group for 24 weeks, and telephone consultation. In DBT-A the skills training group is delivered jointly with adolescents and parents participating together. In a recent review (16) of skills training groups with duration less than 24 weeks, the authors concluded that even at a reduced length, DBT-A skills groups still have clinical utility for several clinical symptoms.

During the last decade, other emotion regulation interventions for adolescents have been developed, such as Emotion Regulation Individual Therapy for Adolescents [ERITA (17, 18)] consisting of 12 weeks treatment, for example, and Unified Protocol for Adolescents [UP-A (19)] for approximately 16 weeks.

Strategies for regulating emotions continue to evolve during childhood and adolescence. Adolescence is a transitional period in life with many biological, emotional, and cognitive processes developing simultaneously, together with changes in the social environment, family and peer relationships (20, 21). Developmental changes occur in the brain during this period and executive control processes, such as inhibitory control systems, are still maturing (22). These developmental challenges and the risk of developing psychiatric conditions during adolescence (23) highlight a need for targeting emotion regulation in psychological treatments during adolescence.

Parents can be suitable role models for adaptive emotion regulation strategies, and parents can also inadvertently maintain maladaptive strategies in children, for example by helping their child avoid negative emotions (24). Focusing on gradually removing such parental accommodation has proved successful in the treatment of childhood anxiety, for example (25). Research has shown that when parents support the child’s emotional expression, the child’s ability to regulate emotions improves (24, 26). There are thus potential benefits for adolescents and parents to learn and practice emotion regulation skills together, which has been recognized in DBT-A, for example.

Despite the promising effects of targeting ER difficulties, the teaching of emotion regulation skills is not, however, routinely and systematically implemented in clinical child and adolescent psychiatric (CAP) practice, and not accessible to everyone. Difficulties in getting families to sign up for and complete extensive treatment programs and a lack of therapists with the necessary training are potential hindrances. Hence, many adolescents and their parents are not given the opportunity to learn adaptive strategies for emotion regulation.

Treatments focusing on ER skills for adolescents are comprehensive and extensive, such as DBT-A and UP-A. There has been a lack of brief interventions focusing on psychoeducation on emotions and ER skills in CAP practice. There is little knowledge of how such brief interventions are perceived by adolescents and parents when delivered jointly, if they are acceptable and whether they have an effect. The transdiagnostic ER group skills training described in the current study is an add-on treatment in a CAP outpatient setting.

A previous pilot study of a five-session version of this skills training showed preliminary promising results on quantitative outcomes, such as ER difficulties and alexithymia, for example (27). However, results need to be replicated in randomized trials with control conditions. A qualitative analysis of 21 interviews with participants who had taken part in the extended seven-session ER group skills training described here focused on process-related variables. Results showed that the participants considered the improved parent-child relationship as the main outcome. The group format and the fact that the adolescents and parents participated together were interpreted as facilitators for change. ER skills, behavioral change and knowledge were interpreted as both mechanisms of change and outcomes (28).

Here we describe the content and components of the seven-session transdiagnostic emotion regulation group skills training delivered jointly to adolescents and parents in a CAP outpatient setting. After describing the content of the skills training, we present both quantitative and qualitative data on participants’ perceptions and evaluation specifically of the methodological aspects of the training. Further, we discuss therapeutic practices, challenges, improvements and recommendations in clinical settings based on participants’ feedback. The current study aimed to examine participants’ perceptions and acceptability, specifically of the methodological aspects of the ER group skills training in a CAP outpatient setting with the following research question: What were participants’ perceptions and acceptability of the methodological aspects of the ER group skills training?

## Method

The study is part of a randomized controlled trial (RCT) of an emotion regulation skills training for adolescents and parents. The setting was two CAP outpatient clinics. The current study included quantitative and qualitative data. Semi-structural interviews were also included.

### Participants

The skills training was transdiagnostic and open to patients 14 – 17 years old and their parents. Exclusion criteria were current substance abuse, severe anorexia, schizophrenia, and ongoing psychosis. A total of 119 participants (56 adolescents and 63 parents) had completed the skills training (this study included participants both from the active group and the control group, who received the treatment at a later stage) by the time of the current study and were asked to evaluate the treatment at the end of the last session. In total there were 20 skills groups with a mean of 4.95 (*SD* = 1.88) families per group (range 2 – 10). In the current study sample, 99 adolescents were randomized, 78 began the intervention and 56 took part in the last session. For demographic information on the 56 adolescents, see **Table 1**. Of the 63 parents that participated, 39 (61.9%) were female, 21 (33.3%) were male and three (4.8%) had missing data. All but one parent was born in Sweden, and 39 (62.9%) had a university/college education.

**Table 1:** Adolescents’ demographics (n = 56).

Variables	Frequency (%)
Females Males	49 (87.5) 7 (12.5)
Age baseline, *m* (*sd*)	16.1 (1.34)
DSM-5 diagnoses*		
Anxiety disorders	47 (83.9)
ADHD/ADD	37 (66.1)
Depression	34 (60.7)
High-functioning autism	8 (14.3)
PTSD	4 (7.1)
Sleep disorder	4 (7.1)
OCD	3 (5.4)
Eating disorder	3 (5.4)
ODD	2 (3.6)
Number of diagnoses		
1	14 (25.0)
2	16 (28.6)
3	15 (26.8)
4	7 (12.5)
≥ 5	4 (7.1)
Parents born outside of Sweden		
Mother	6 (11.0)
Father	4 (7.0)

*Each participant could have several diagnoses. DSM-5, Diagnostic and Statistical Manual for Mental Disorders, fifth version; ADHD, Attention Deficit Hyperactivity Disorder; ADD, Attention Deficit Disorder; PTSD, Post-Traumatic Stress Disorder; OCD, Obsessive Compulsive Disorder; ODD, Oppositional Defiant Disorder.

Of the total 119 participants, 117 (98.3%) (54 adolescents, 60 adults and three individuals with missing data on adolescent/parent status) submitted quantitative evaluative data at the end of the last session and 92 (77.3%) participants (35 adolescents and 55 parents and two individuals with missing data on adolescent/parent status) submitted qualitative evaluative data.

The selection of participants for interviews was made to maximize the variation in the sample and included both adolescents and adults in different family constellations, of different genders and ages, as well as participants who had dropped out and those who had completed the intervention from both CAP clinics and with different skills trainers. To be eligible for an interview, participants had to attend at least one session of skills training during 2021. Based on these criteria, skills trainers were instructed to give suggestions to maximize the variation. This resulted in 38 participants. The first author then reviewed eligible participants for variation and approached 26 participants by telephone. Of these, one did not answer and four declined, resulting in a total of 21 participants for interviews. Of these, 11 (seven women and four men) were parents, and 10 (nine girls and one boy) were adolescents. Nine adolescents and 10 parents were related. In one family, two parents were interviewed. One adolescent and one parent in the sample were unrelated. The interviewed participants attended four to seven sessions, *M* = 6.30, median = 7. See also Holmqvist Larsson et al. (28) for more information on participants’ experiences.

### Procedure

Participants were assessed with the Mini International Neuropsychiatric Interview for Children and Adolescents [M.I.N.I KID 6.0 (29)], for psychiatric diagnoses as part of an initial assessment. After the treatment, participants were asked to anonymously evaluate the treatment by answering nine statements/questions developed for this study at the end of the last skills training session. Answers were coded in fixed categories using a Likert scale ranging from 1 “no, not at all”/”very bad” to 5 “yes, very much”/”very good”, with one answer ranging from 1 “no”, 2 “maybe” and 3 “yes”. Eighty-one written comments (of which 46 comments were from parents and 35 from adolescents) were submitted to the question “Do you think that the skills training has and/or will be able to contribute to a better relationship between yourself and your family members?” Fifty-nine (43 from parents and 16 from adolescents) comments were submitted when participants were encouraged to leave general comments of their experience of taking part in the skills training. In total, 140 written comments from 92 participants were included in the current analysis together with semi-structured interviews from 21 participants.

Due to the pandemic, 10 interviews were held digitally, 10 by telephone and one face-to-face during the period May 2021 – February 2022. Adolescents and parents were interviewed individually and separately. Interviews were conducted 3-4 months after the skills training by two female psychotherapists trained in DBT and with experience of clinical CAP. One of the interviewers (the first author) had been involved in the recruitment process to the RCT, but beyond this, no interviewer had any prior relation to the interviewed participants. The interviews were semi-structured with open questions about the experiences of participating in the skills training. Mean length of interviews was 20 minutes (varying between 8-38 minutes). Interviews were recorded and transcribed verbatim.

Skills trainers were master students in psychology who were training for their clinical psychologist license, and licensed psychologists and/or licensed psychotherapists. Senior and junior skills trainers always held the skills training together. All skills trainers had CBT-training and clinical experience from CAP and were trained by the first author. The emotion regulation skills training was delivered as an adjacent, add-on treatment, in a CAP setting.

### Intervention

The overall aim of the skills training is to reduce difficulties with emotion regulation and psychiatric symptoms and increase emotional awareness and quality of life. The conceptual model has its origin in acceptance-based behavioral therapies and focuses primarily on behavioral change, mindfulness, and acceptance with particular emphasis on communication skills and validation, in a family context of CAP. The emotion regulation group skills training that is presented here is inspired by third-wave evidence-based cognitive behavioral therapy (CBT) with emotion regulation as one of the core components, such as Dialectical Behavior Therapy [DBT (11)], Emotion Regulation Group Therapy [ERGT (30)], Unified Protocol [UP (31)], and treatment principals from Acceptance and Commitment Therapy [ACT (32)], CAP practice and guidelines, as well as neuroscientific findings that support the importance of a good so-called body balance and high levels of emotional granularity (33).

The model is based on the theory that postulates that dysregulated emotions drive impulsive behaviors, psychiatric symptoms, and suffering. The conceptualization of emotion regulation in the current intervention is in line with the definition of emotion regulation by Gratz and Roemer (5), which emphasizes awareness, understanding and acceptance of emotions. The skills training aims at giving participants an increased awareness and knowledge of what emotions are and how emotions work; understanding the functions of emotions; increasing skills in identifying and labeling emotions; expressing primary emotions and self-validation; regulating emotions and accepting emotions and acting in accordance with their valued direction, even in moments of strong emotional arousal, and reducing emotional vulnerability. See **Table 2**.

**Table 2:** Overview of the content of the emotion regulation skills training.

Sessions	Content
Session 1	Awareness of emotions and labeling emotions
Session 2	Identifying emotions and the functions of emotions
Session 3	Primary and secondary emotion. Validating and reducing judgment
Session 4	Reducing vulnerability and emotional imbalance
Session 5	Making conscious choices – goal directed behaviors
Session 6	Acceptance and valued directions
Session 7	Repetition
Booster session	Repetition and maintenance of skills

The course format is seven two-hour, weekly sessions, including a booster session after three months. Each session follows the same structure: repetition of the content from the previous session, review of homework assignments from the previous session, and a break with snacks before the new theme and skills are presented, which results in a new homework assignment. The skills training is led by two skills trainers. PowerPoints are used as pedagogical tools, as are videoclips of films and pictures, group discussions, exercises, and role plays. Participants receive a handout version of the PowerPoint at each session.

#### Session 1: introduction, awareness of emotions and labeling emotions

The aim of Session 1 is to increase awareness of emotions, to become curious about one’s internal and emotional life, and to recognize, identify and label emotions. Session 1 begins with psychoeducation about what emotions are, what emotions are not and the importance of differentiating between specific emotions, as well as identifying and labeling emotions, and why it is important to be able to distinguish between thoughts, emotions, and behaviors. The term emotion regulation is explained. Examples of consequences of emotion dysregulation are presented, as are the short and long-term consequences of avoiding emotions. Participants are introduced to an exercise in emotional awareness. Film clips are shown from popular culture where people experience and display different emotions, body language, impulses, emotional expression, and avoidance strategies. Participants are encouraged to describe the emotions they see being displayed by others in the film clips and thereafter to prompt their own emotional reactions to practice identifying and labeling emotions. A brief and basic psychoeducation of the neuroscience behind the importance of labeling emotions is also presented (33), as is an evolutionary perspective of the purpose of emotions. The skills trainers describe more general and less specific expressions of emotions and discuss the consequences of using vague rather than more specific emotional language. The group works with a practical exercise to distinguish between emotions, thoughts and actions. The skills taught and practiced in this session are emotional awareness and identifying and labeling emotions. The homework assignment for Session 1 is to practice emotional awareness, by identifying and labeling emotions and identifying actions in an everyday situation. A worksheet for this purpose is given to the participants.

#### Session 2: identifying emotions and the functions of emotions

Session 2 focuses on the functions of emotions and skills to identify emotions. The participants are taught that the function of the emotion provides us with important information. Psychoeducation about the impulses and functions of emotions is provided, and the group works collaboratively in a group exercise to identify impulses/actions and functions connected to specific emotions. Session 2 also focuses on skills for identifying emotions. For each skill that is taught, participants are engaged in in-sessions exercises. As a practical exercise, the skills trainers give examples of different behavioral impulses, and the participants try to identify associated emotions. Throughout the whole session it is emphasized that it is important to listen to, and take care of, the need embedded in the identified emotion. The skills taught and practiced in Session 2 are identifying emotions, and learning to take care of the needs embedded in the emotions, such as grieving and needing to be comforted when experiencing sadness. The homework assignment for Session 2 is the same as the previous week, with the addition that the participants also register the impulse and the function of the emotion that they identify in a specific situation.

#### Session 3: primary and secondary emotion, validation of self and others

Session 3 focuses on primary and secondary emotions, validation and reducing judgment. This session is inspired by Marsha Linehan’s description of primary and secondary emotion and validation as presented in DBT (11, 34). Participants are taught that the knowledge about primary and secondary emotions can be helpful in understanding how negative emotions are prolonged and exacerbated. Participants are also taught how and why secondary emotions arise and learn to distinguish between primary and secondary emotions. Secondary emotions typically arise when our first emotional reaction to an event (the primary emotion) is avoided or judged. Blaming oneself for reacting with sadness, for example, can result in secondary shame. Explanations of why it is common that primary emotions are missed or avoided are presented and the potential challenges and negative consequences of secondary emotions are explained and discussed. The skills trainers use concrete, everyday examples to illustrate this, and participants take part in exercises to practice the skill of differentiating between primary and secondary emotions. The session continues with psychoeducation about validation and judgmental thoughts. When we validate ourselves, we let ourselves know that our inner experiences, reactions, thoughts or emotions are valid, i.e., they make sense, which can be helpful in reducing strong emotional reactions. Participants engage in an in-session exercise about self-validation and judgmental thoughts. Participants are encouraged to come up with examples of judgmental and self-validating thoughts in response to different situations, and the potential consequences of these thoughts are discussed. Psychoeducation about how to express primary emotions is taught as part of facilitating validation. Parents practice validation skills and adolescents practice the skill of expressing primary emotions in an in-session role play exercise. Skills taught in Session 3 are: distinguishing between primary and secondary emotions, expression of primary emotion, and validation of both self and others. Adolescents and parents receive a separate homework assignment for this session. Adolescents are given an assignment to practice self-validation and parents practice validating their child.

#### Session 4: reducing vulnerability and emotional imbalance

The aim of Session 4 is to reduce vulnerability and emotional imbalance. The skills trainers give the rationale for reducing emotional vulnerability. This session focuses on both reducing vulnerability and increasing stability. The participants are given psychoeducation on the importance of routines for sleep, food and physical activity and its connection to mental health in general and emotional imbalance. In a group exercise, the participants discuss what they are most strongly affected by: lack of sleep, lack of routines for eating or lack of physical activity, for example. The participants work on problem-solving together and discuss what would make it easier to make minor changes. The exercise is rounded off by going through the skill of increasing positive activities. Recovery is also addressed to increase well-being and reduce emotional imbalance. The importance of finding a balance between stress/demands and recovery/rest to increase stability is described. Skills taught in Session 4 are: reducing vulnerability by taking care of routines for sleeping, eating, and physical activity, as well as increasing positive experiences and balancing the need for rest and recovery. The homework assignment for Session 4 is to schedule two physical or positive activities for the week and register reflections when they are done. Adolescents and parents are encouraged to do the activities together.

#### Session 5: making conscious choices – goal-directed behaviors

Session 5 focuses on behavior and how to engage in goal-directed behaviors when experiencing emotions, rather than acting impulsively. This session is partly based on Linehan’s concept of how to change emotional responses as described in DBT (11, 34). It is explained that one of the fundamental aims of emotion regulation is to be able to act in a goal-directed way despite the presence of negative emotions. The skill “pause” is introduced and different techniques for making it possible to take a pause are discussed, to give oneself the opportunity to make a conscious behavioral choice. Three behavioral choices are presented to the participants: to act on the emotion’s impulse, to act in the opposite way of the emotion’s impulse and to remain with the emotion without acting on it. To be able to make conscious decisions for our behavior, it is necessary to first identify the emotion, label it, determine if it is a primary emotion or not, self-validate, “pause” and thereafter make the choice of how to act, based on what consequences the behavior will have. The importance of taking care of the needs in the emotions, regardless of which behavior we choose, is also emphasized. An in-session exercise that focuses on different behavioral responses to different emotions and situations is presented to increase the practical understanding of the difference between the different choices. The skills that are taught in Session 5 are: to pause and consciously choose a behavioral response. The homework assignment for this session is to choose a situation with an emotional reaction and register what behavior was chosen and which consequences the behavioral choice had.

#### Session 6: acceptance and valued directions

Session 6 focuses on acceptance and valued directions. This session is inspired by ACT (35) and uses Hayes’ definition of acceptance as “the active and aware embrace of private experiences without unnecessary attempts to change their frequency or form.”(32, p. 982). A rationale is given to the participants on the concept of acceptance and what it involves. ACT metaphors are used to illustrate acceptance. It is emphasized that emotions are amplified when we try to avoid them. Openness is introduced as a skill to be able to accept. Examples of how we can use our body to increase openness are taught, such as: unclenching the fists, raising the eyes, relaxing the facial muscles and dropping the shoulders. Session 6 continues by focusing on valued direction. The concept of values and valued direction is introduced. Exercises that aim at encouraging the participants to think about what they value in life are presented.

The skills taught and practiced in Session 6 are: acceptance, openness, values and valued direction. The homework assignment is an adaptation of the life compass from ACT (35), where the participants practice identifying their values by grading different areas in life (such as family, friends, education) ​​and prioritizing how important they are to them. This assignment is not reported to the other participants in the next session. Instead, a joint discussion on what it was like to begin working on life values is initiated.

#### Session 7: repetition

Session 7 focuses on repeating the content of the previous sessions, focusing on the skills that have been taught and exercises. A written summary of the emotion regulation skills is distributed to the participants and all the skills are repeated during this last session. Three months later a booster session takes place, with similar content to Session 7. The booster session also focuses specifically on generalization of skills.

### Data analysis

#### Quantitative analysis

The quantitative descriptive data were presented using frequencies and percentages. Between group differences (adolescents vs. parents) were analyzed with crosstab analysis with effect size of Cramer’s V and Phi for 0.07, 0.21 and 0.35, and 0.1, 0.3, 0.5 for small, medium and large effect, respectively.

#### Qualitative analysis

The semi-structured interviews and written comments were analyzed using thematic analysis, to identify, analyze and present patterns or themes within the data, using the six steps described by Braun and Clarke (36, 37). Our orientation to data on the deductive and inductive continuum had a deductive approach, focusing specifically on the evaluation and acceptability of the methodological aspects of the intervention, and also took on an inductive approach where analysis was based on data and opened up to more meaning-based interpretation. Data was analyzed on a semantic/explicit level to keep close to the participants’ expressions. Codes were produced individually by the two authors, and themes were created, labeled, and discussed together. Both authors are female and CAP-clinicians (licensed psychotherapists with CBT orientation) with experience of working clinically with children and families and emotion regulation skills training, and research focusing on child and adolescent mental health.

## Results

### Quantitative analysis of participants’ experiences

The quantitative evaluation showed that a majority of both adolescents and parents were positive to the skills training and 94.0% of the total sample would recommend it to others (**Table 3**). Parents’ and adolescents’ ratings were compared using crosstabs analysis. Since there were so few negative responses, the two negative response options were collapsed into one and the two positive response options were collapsed into one, resulting in three categories for the analysis: positive, neutral and negative. Although adolescents’ ratings generally were positive, more parents rated that the skills training was good/very good (98.3% vs 85.2%), χ^2^ = 4.95 (1), *p* = .03, Phi = .24, indicating a small effect. More parents would recommend the skills training to others (100.0% vs. 87.0%), χ^2^ = 8.29 (2), *p* = .02, Cramer’s V = .27, medium effect, and rated that the skills training contributed to a better relationship with family members (83.3% vs. 59.3%), χ^2^ = 8.99 (2), *p* = .01, Cramer’s V = .28, medium effect. On the family relationships item, 83.3% of parents and 59.3% of adolescents answered yes, while 37% of adolescents rated “maybe”. All other comparisons between parents and adolescents were non-significant. Of parents, 91.6% reported that the skills training had made them better equipped to cope with problems compared to 77.8% of adolescents. Parents and adolescents rated that they had learned more about emotions (93.3% vs. 90.7%), learned to recognize emotions (91.7% vs. 81.5%), learned more about the functions of emotions (91.7% vs. 88.9%), and could deal with their emotions better now than before the skills training (75.0% vs. 66.7%). On this last item, 23.3% of parents, and 22.2% of adolescents, rated a “neither/nor” response. See **Table 3**.

**Table 3:** Participants’ evaluation of a joint emotion regulation group skills training, frequencies and percentages.

Items	Total *n* = 117*	Adolescents *n* = 54					Parents *n* = 60					Stats.≈
		Positive		Neutral	Negative		Positive		Neutral	Negative		
	yes/good and yes, very much /very good’	yes, very much/ very good	yes/good	neither nor	no/bad	no, not at all /very bad	yes, very much/ very good	yes/good	neither nor	no/bad	no, not at all /very bad	
	*n* (%)	*n* (%)	*n* (%)	*n* (%)	*n* (%)	*n* (%)	*n* (%)	*n* (%)	*n* (%)	*n* (%)	*n* (%)	*P*
Considering the whole skills training, what did you think?⁑ ˜	107 (92.2)	30 (55.6)	16 (29.6)	8 (14.8)	0 (0)	0 (0)	38 (64.4)	20 (33.9)	1 (1.7)	0 (0)	0 (0)	.03
Has the skills training made you better equipped to cope with your problems?	99 (84.6)	10 (18.5)	32 (59.3)	10 (18.5)	1 (1.9)	1 (1.9)	23 (38.3)	32 (53.3)	4 (6.7)	0 (0)	1 (1.7)	.11
Would you recommend the skills training to others?	110 (94.0)	31 (57.4)	16 (29.6)	6 (11.1)	1 (1.9)	0 (0)	52 (86.7)	8 (13.3)	0 (0)	0 (0)	0 (0)	.02
Do you think that the skills training has and/or will be able to contribute to a better relationship between yourself and your family members? ‡	84 (71.8)		32 (59.3)	20 (37.0)	2 (3.7)			50 (83.3)	10 (16.7)	0 (0)		.01
Do you think that the skills training has increased your capability to deal with difficult life situations?⁑	93 (80.2)	7 (13.0)	32 (59.3)	13 (24.1)	1 (1.9)	1 (1.9)	13 (22.0)	38 (64.4)	7 (11.9)	0 (0)	1 (1.7)	.17
I have learnt more about emotions	108 (92.3)	21 (38.9)	28 (51.8)	3 (5.6)	1 (1.9)	1 (1.9)	32 (53.3)	24 (40.0)	3 (5.0)	1 (1.7)	0 (0)	.78
I have learnt to recognize emotions	100 (85.5)	19 (35.2)	25 (46.3)	9 (16.7)	1 (1.9)	0 (0)	25 (41.7)	30 (50.0)	4 (6.7)	1 (1.7)	0 (0)	.24
I have learnt more about the functions of emotions	106 (90.6)	19 (35.2)	29 (53.7)	5 (9.3)	1 (1.9)	0 (0)	31 (51.7)	24 (40.0)	4 (6.7)	1 (1.7)	0 (0)	.87
I can deal with my emotions better now than before the skills training	82 (70.1)	11 (20.4)	25 (46.3)	12 (22.2)	5 (9.3)	1 (1.9)	13 (21.7)	32 (53.3)	14 (23.3)	1 (1.7)	0 (0)	.11

* Three participants had missing data on adolescent/parent status. ‘Response options yes/good and yes, very much/very good were collapsed for presentation of total sample in this column. ⁑One parent missing, n = 116, n = 59. ‡Three response options: yes, maybe, no. ≈ Chi-square analysis 3 x 2 comparing parents and adolescents (2) on collapsed positive (yes, very much/very good and yes/good), negative (no, not at all/very bad and no/bad) and neutral (neither nor) response categories (3). ˜Crosstab analysis conducted for 2 x 2 categories since there were no negative responses to this statement. Analyses were also run with Fisher’s exact test due to small sample size in the negative response options, which didn’t change significant results.

### Qualitative analysis of participants’ experiences

Analysis of 21 semi-structured interviews and 140 written comments from 92 individuals resulted in three overarching themes: Treatment components, the learning climate and Pedagogical aspects, with several subthemes. See **Table 4**.

**Table 4:** Overarching themes and themes.

Overarching themes	Themes
Treatment components	Focusing on skills
	One size doesn’t fit all
The learning climate	Therapeutic approach
	Being together with others
	The question of susceptibility
Pedagogical aspects	Group constellation: differences and similarities
	Too little or too much
	Variety stimulates learning

#### Treatment components

Participants appreciated focusing on emotions and skills training in treatment and identified emotions as a crucial component in relation to their well-being. They described positive aspects of the skills training and mentioned specific skills that were valuable to them, as well as challenges. This was analyzed as: Treatment components and was related to the content of the treatment. Two subthemes were identified: Focusing on skills and One size doesn’t fit all.

Focusing on skills: Several participants described that emotion regulation skills are important life skills that everyone should know, as it increases understanding of oneself and others: *“A good and important course that everyone should do!”* (parent #26). The skills that were most often commented on as being especially helpful were identifying primary emotions: *“I’m more conscious of how my secondary emotions rule my life and have taken over my behavior in a negative way - with this group I’ve started to notice and to ask myself what I am feeling and I dare/am able to discuss it with other family members”* (parent #35), validation: *“For regardless of whether I have got more control over my feelings, more people should get more knowledge, and especially about validation, so that more people can understand and help those who get extreme in their feelings”* (adolescent #23), and combining these two skills: *“Getting tools to identify the primary emotion so that I can validate/put into words my daughter’s emotion”* (parent #22).

Other skills that were mentioned by participants were to stop and take a pause before acting on an emotion: “*To stop for a moment, think about what you were feeling and why. When I learned that I got more control over myself and the situation”* (interview, adolescent #2) and to reflect and take an interest in one’s inner world: *“That you are a bit more curious, why do I do this, and this moved me and made me sad…”* (interview, parent #1). Accepting emotions, taking care of the need embedded in the primary emotion and practicing self-validation were also mentioned: *“That you in fact dare admit to yourself that, no, maybe it’s actually fear I’m feeling now … and then take care of it”* (interview, parent #5).

One size doesn’t fit all: Many comments were related to the level of difficulty but how this was perceived varied somewhat between participants. Some mentioned that the content was difficult, somewhat academic in nature and a lot to take in: *“But I think that for some it might kind of feel too difficult, just because of the language, like, that it’s too difficult”* (interview, parent #2). Others thought that especially the first session was perceived as too basic: *“The main theme became clearer, I would say, on the second session but it was a bit difficult to see it the first time because it felt very basic, maybe a bit too basic”* (interview, parent #7).

Several participants described that it took some time before the content sank in. Some experienced that the big picture began to fall into place after the first two sessions: *“I thought the course was instructive but you have to give it a chance, since the first times weren’t all that much fun, but in the end you take away a lot”* (adolescent #48*).*

The skill reducing vulnerability by increasing routines for food, sleep and physical activity generated the most varied comments where some especially appreciated this session, while others thought that it was unnecessary. Participants also expressed what content they lacked in the skills training. Some wanted less focus on what emotions are and more strategies on how to cope with difficult emotions. Some participants requested more specific content on how to cope with anxiety.

#### The learning climate

Several of the participants’ comments were related to the group format and the skills trainers. Participants also discussed the timing of participation and the challenges of engaging the adolescents. This was analyzed as The learning climate and contains process-related experiences. Three subthemes were identified: Therapeutic approach, Being together with others, and The question of susceptibility.

Therapeutic approach: Participants spontaneously commented on and praised the skills trainers as being an important contributing factor in how the emotion regulation skills training was perceived. Comments were generally very positive, and it was the way the skills trainers led the training that was emphasized. It was especially appreciated that the skills trainers seemed to enjoy leading skills training, were easy going, transparent, engaged and enthusiastic, and used a lot of humor: *“Good and committed teachers who give the best of themselves and their expertise”* (parent #38). The interaction between the two skills trainers was also commented on as an important factor for successful delivery of the content. Furthermore, self-disclosure seemed to be especially appreciated and was commented on by several participants: *“They brought up a lot of things themselves, like, own examples, and I thought that was very good”* (interview, adolescent #1).

Being together with others: Overall, both parents and adolescents expressed that it was valuable to participate together in the skills training. The advantage of learning a mutual language for emotions was emphasized: *“Very good with parents and youngsters together → more fun and broader discussions”* (parent #13). Parents and adolescents thought that it was interesting to hear one’s family member talk about thoughts, emotions, and difficulties in regulating emotions: *“I think that being able to see and validate each other’s emotions will contribute to a deeper relationship”* (adolescent #44). Some adolescents also expressed that it would have been beneficial if one of the sessions was separate, as it would have been easier to talk about certain things if the parents were not present. This was also commented on by a few parents. One process aspect that was mentioned by some participants, mainly by parents, was that adolescents tended to be passive initially and appeared non-engaged. Several adolescents suffered from depression and social anxiety, for example, and some had high-functioning autism, and the group format was initially challenging for some.

Participants were generally appreciative of the group format which had several advantages. Both the skills trainers and other group members contributed to a safe environment*: “I loved the Group and I wish I had talked more and not been so shy”* (Adolescent #34). Some participants reported that the sessions would be missed and that they looked forward to the sessions: *“I actually looked forward to going to the emotion regulation the days that we were there”* (interview, adolescent #8). Some participants, however, also expressed a need for more individual adjustment that wasn’t possible in the group format.

The question of susceptibility: Participants mentioned the timing aspect, i.e., when the skills training was delivered in relation to other treatment interventions, adolescents’ symptom severity or general level of functioning. Several stated that the skills training would have been helpful earlier on in treatment. At the same time, the skills training was perceived as too demanding if symptom severity was too high and some argued that the course would have been better if it had been given at a later stage in treatment: *“Now he would have been receptive in a completely different way than he actually was then, then it was a struggle, like, just to stay alert for two hours during the course”* (interview, parent #2). Unmedicated depression and ADHD were mentioned as examples of conditions that made it difficult for the adolescent to utilize the content: *“It has actually helped her, but it would have helped her a hundred times more if she had had some medication at the time…”* (interview, parent #8). There thus seemed to be a middle path, where the skills training is delivered when the adolescent is a little more stable: *“I thought it was quite good that it came now when things are a bit better”* (interview, adolescent #3). Some participants mentioned on a more general note, that the emotion regulation skills training ideally should be given earlier in the adolescent’s life, as a preventive intervention. Participants wondered why they hadn’t been taught it earlier: *“So incredibly good! Parents in general need this, kids need to know this at an early age”* (parent #24).

#### Pedagogical aspects

Participants commented on the length of sessions and treatment, the group constellation, and homework, which was analyzed as Pedagogical aspects. This theme relates to the structure of the skills training and includes three subthemes: Group constellation: differences and similarities, Too little or too much, and Variety stimulates learning.

Group constellation: differences and similarities: Some reflections were related to the transdiagnostic inclusion of participants. Participants noted that they had a lot in common, and shared challenges related to difficulties with regulating emotions despite having different diagnoses. The transdiagnostic group composition worked well and was in general received positively. A few participants mentioned some difficulties, however, especially for those with high-functioning autism: *“The content has been very good. Sometimes it can be difficult to identify with something, when/if you have an autism diagnosis. Shame, for example, hard to see why you would want to belong to a group”* (parent #60). There were also comments, however, that the skills training was valuable for those with high-functioning autism: *“I see a big difference in [adolescent], who has begun to say what emotion she has and at the same time she asks me and others how we are feeling when she can’t ‘read’ us”* (parent #45). Concerning the group constellation, several voices were raised about the lack of male participants, referring to both adolescents: *“Could have wished for a more even distribution between the sexes of the youngsters”* (parent #48) and parents*:”…It could be worth thinking about equality … try to coach more dads to go”* (parent #33). Some groups consisted of only a few participants, which was generally perceived as something positive. Some suggested having different groups for younger and older adolescents, as adolescents had different cognitive and emotional abilities.

Too little or Too much: The length of the sessions was commented on by participants and the two-hour sessions were perceived as long by several adolescents: *“The sessions were a bit too long, you got too tired”* (adolescent #53). Adolescents also commented on it being difficult for them to concentrate during the entire session: *“… It’s difficult to sit still for that long and just listen, you lose your concentration after a while”* (adolescent #27). Concerning the number of sessions, participants’ experiences varied. Some commented that seven sessions were too many, while others mentioned that there were too few sessions and that they needed more time to practice: *“Very good course, but it takes time and training to use what you have learned” (*adolescent #46). On the other hand, some participants mentioned that many sessions took time from work and school. Some suggested having more time between sessions toward the end of the skills training to allow more time to practice. Comments relating to the structure, and length and number of sessions were the most heterogeneous.

Variety stimulates learning: One subtheme under the theme Pedagogical aspects was related to the variety of the skills training. Participants appreciated the mix of PowerPoints, films, role play, lectures and receiving hand-outs: *“It has been really good and very clear with lectures and images, handouts and films”* (parent #55). The film sequences were especially appreciated, adding humor and making difficult topics easier. Several participants mentioned that they appreciated the way the skills training was structured: “*Good setup. I’ve previously been in a lot of therapy and have taken away a lot of what was taken up in the course, but here it was clearly laid out and I can see/understand more how things fit together”* (parent #25). Several participants requested more practical exercises and film clips, and also more time for discussions, reflection, and more examples from everyday life on how to apply the knowledge. This was also emphasized in relation to the need to adjust the pedagogy to the adolescents to increase their endurance and motivation: *“Maybe a few more activities or whatever you call it, there was, like, a lot of sitting still and a lot of talking”* (interview, adolescent #4).

Homework was generally commented on as important and necessary, and as an opportunity to practice the skills: *“The homework assignments meant that I, like, understood why I needed to have this”* (interview, adolescent #11). Some mentioned that they presented the homework to other family members who did not participate in the skills training. Several participants, however, problematized that it was difficult to remember the homework: *“We were given homework every time which I thought was good, but we forgot it every fricking time … we did it, like, in the car on the way there every time…”* (interview, parent #9) and suggested that it would help to get a reminder.

## Discussion

A seven-session transdiagnostic cognitive behavioral approach to regulating emotions in a joint skills training format for adolescents and parents in a CAP outpatient setting was described. The acceptability of the method was evaluated based on quantitative evaluations from 117 participants, and qualitative analysis of 21 interviews and 140 written comments. Results from the quantitative evaluation showed that both adolescents and parents were generally positive about the skills training and would recommend it to others. The three main themes that were generated in the qualitative analysis describe participants’ experiences of the content, process and structure of the skills training. Different therapeutic implications and recommendations for treatment practices are addressed and discussed below.

### Treatment components

Focusing on skills and ER as an underlying construct in treatment was generally perceived as positive and meaningful. Participants recognized that emotions were at the core of several of their difficulties and influenced their everyday life. Psychoeducation about emotions, their function and evolutionary purpose, and learning emotion regulation skills were perceived as valuable and important. With the third wave of CBT emphasizing flexibility and a broad repertoire of behaviors over the reduction of symptoms and narrowly defined problem behaviors (35), several treatments have included ER as a core component, such as DBT, ERGT and UP (11, 30, 31). Several of these methods have shown successful outcomes in treatment research (7, 8, 14, 17, 38, 39) and ER is often found to mediate treatment effects (40–42). In addition to these quantitative outcome studies, our results on the importance of emotions confirm earlier qualitative research where participants experience that focusing on emotion regulation in treatment is meaningful and helpful (43, 44). The concept thus seems to have high social validity for participants, and focusing on emotions for treating behavior problems and psychiatric symptoms seems relevant and appropriate for participants. Targeting emotion regulation can thus be beneficial when treating adolescents since emotion regulation is an essential developmental task and is related to mental health and behavioral problems (45).

Where specific ER skills were concerned, identifying primary emotions and differentiating between primary and secondary emotions were seen as especially helpful and described by some as an “Aha” experience that opened their eyes to this phenomenon. Parents also reported that validation was an especially useful tool in relation to their children. Validation is an important communication tool (46), which can promote effective emotion regulation (47). Validation can also be a necessary prerequisite when trying to motivate patients to behavioral activation and exposure, for example, especially for adolescents who can perceive too much focus on change as criticism of their current choices and behaviors. Focusing on emotions and validation can therefore facilitate change.

Participants were initially somewhat confused about the purpose of the skills training and how it would all tie together. In the first session, skills trainers explain that the skills training is like a puzzle, and that adding one piece at a time will eventually lead to the bigger picture. Based on participants’ comments, this information is important and can become clearer. Participants are probably occupied with several processes initially and this message therefore needs repeating throughout the training. It is also important that skills trainers trust the process, even if participants are hesitant initially.

Concerning the actual content, one size doesn’t fit all. Some participants wanted more information and skills on how to reduce vulnerability, for example, while others perceived this as unnecessary. Several adolescents who suffer from mental health problems, especially individuals with neurodiversity, can experience difficulties with establishing and maintaining routines for sleep, food, physical exercise and positive activities, as well as balancing stress and rest (48, 49), making them more emotionally vulnerable and potentially less well-equipped to face life challenges (50). Reducing vulnerability is a central part of mental health, in line with Barrett’s concept of balancing a body budget (33), and Linehan’s terminology of building a resilient biology (34). This is an important prerequisite for regulating emotions and therefore has a place in ER skills training.

Furthermore, some reported that the content was too basic, while others perceived it to be very complex and difficult. There are challenges in a group format in addressing individual differences and needs. Participants have different intellectual capacities, levels of functioning, symptoms, and behavior problems, as well as learning profiles. The most optimal approach is probably to teach emotion regulation skills in groups and offer more individualized treatment in combination, if needed, based on respective family’s needs.

### The learning climate

Skills trainers were encouraged to practice a therapeutic approach that is characterized by a relaxed and easy-going manner, to use warmth and humor and model the skills during the sessions. The skills trainers therefore need to share their own experiences, thoughts, emotions, and behaviors with participants. Such an approach is recommended for therapists in DBT, for example, for adolescents suffering from BPD (13). This self-disclosure was appreciated by both adolescents and parents in the current study and was perceived as validating. It is thus probably helpful for several different patient groups, in addition to those suffering from BPD. That skills trainers share their own experiences in an easy-going manner, showing that we are all humans who struggle with emotions, could potentially also lead to normalization and less stigma.

Using humor, a personal communication style and an easy-going manner can also be one way of getting adolescents’ attention initially. If skills trainers can break the expectancy that comes with the therapeutic role it can potentially help to engage adolescents. Participants also reflected on when an intervention will have the best effect, for whom the treatment is most effective and when a patient is most susceptible to an intervention. Several participants commented on the timing of participating in the skills training. Timing of strategies, readiness for change and symptom severity of patients are factors that can influence not only acceptability but also outcomes of interventions generally in health care (51, 52). It is therefore important that pros and cons for participating at a given time are weighed against each other during the recruitment phase, given adolescents’ level of functioning and symptom severity. Unmedicated ADHD, for example, or severe depression, can prevent adolescents from benefiting from the content, and could also potentially influence participating adolescents’ perception of the emotion regulation skills training. Here it seemed that the method was most helpful for those not too severely burdened by symptoms that could hinder optimal benefit from the content.

### Pedagogical aspects

Joint sessions for parents and adolescents were appreciated with added benefits being reported, such as increased understanding of each other and gaining a mutual language for emotional communication. Previous research on parental involvement in adolescent interventions has been mixed, but a recent meta-analysis (53) had focused specifically on adolescents found an increased effect on adolescent psychopathology when parents were involved in treatment, compared to interventions that target only adolescents, especially for externalizing symptoms. When treating adolescents and parents together in a joint group format it might be optimal not to have too high a baseline level of family conflict. Based on participants’ comments, one option could be to add one separate session for parents and adolescents, where specific issues related to adolescence or parenthood could be addressed. The transdiagnostic format was feasible for the current sample with several comorbidities, in line with other transdiagnostic approaches that focus on underlying mechanisms rather than a specific display of symptoms (38).

For larger clinics, more homogenous groups, based on age, symptom severity, levels of functioning and cognitive ability, for example, could allow for adjusting and tailoring the length and content to individual abilities and needs. However, heterogeneity and comorbidity are a part of mental disorders in psychiatric care, and transdiagnostic treatments are recommended as one way forward to address this (54).

The group constellation in the current study consisted of mostly female participants and their mothers, which is something that several participants reacted to. Clinicians thus need to try to encourage boys and fathers to participate in group sessions.

The varied pedagogical approach was commented on by participants as positive. In fact, even more role play, discussion, and film clips were requested, especially for the adolescents who needed more variation to keep their attention on the content. The two-hour sessions were perceived by many adolescents as being too long, despite there being a break with snacks. Since the sample was from clinical CAP with several comorbid diagnoses, the skills training could benefit from having even more variation and less reliance on auditive information, for example, in line with special adjustments for participants with ADHD, high-functioning autism and depression, where the possibility to process auditive stimuli and to concentrate for a long period is affected (55). It is a challenge, yet crucial, to keep adolescents engaged despite this.

Homework and between-session activities are an integrated part of CBT (56), and the skills taught were perceived as applicable to everyday life. Despite agreeing with the importance of homework, participants found it difficult to remember and prioritize homework. Since generalization of skills is essential, the importance of homework has to be addressed, and special procedures need to be in place to increase motivation and adherence. One strategy is to use reinforcing contingencies during group sessions (57), where participants receive praise and positive attention for doing homework, and at the same time making it slightly aversive if the homework hasn’t been done. Participants should also be encouraged not to use too sensitive topics so that they can present their homework in front of the whole group. Problem-solving of barriers to completing homework and how these could be overcome should be included. Stimulus control strategies can also be used, such as sending out reminders between sessions (58).

### Limitations

The results need to be interpreted in the light of some limitations. First, the significant differences between parents and adolescents that were found in participants’ evaluation have to be interpreted with some caution due to multiple comparisons.

There could also be a potential bias in those who chose to participate in the skills training and those who completed the whole training and participated in the current study. Families with severe interpersonal problems were perhaps not as likely to participate in a joint skills training. There could also be a bias in which adolescents would consider participating in a group intervention. Those who chose to write comments could potentially have more extreme opinions, both positive and negative. Since the written comments were anonymous, we cannot however examine differences between those who chose to write comments from those who did not. The procedure for recruiting participants for interviews could potentially have contributed to a selection bias. Even though the selection criteria were made to maximize the variation, there could be a bias as to which participants skills trainers suggested.

The sample in the current study was from CAP and recommendations are intended for clinical groups. Further research is needed on other patient samples, in other settings, and by other researchers to confirm the results.

In the qualitative part of the current study, written answers to open-ended questions and transcripts of semi-structured interviews were analyzed together, which could potentially be a limitation, since these methods of collecting data differ somewhat. The former does not allow for prompts or follow-up questions, for example, or the possibility of ensuring that statements have been understood correctly. The text generated from the open-ended questions was also shorter. However, all data were assessed as appropriate to include and analyze to answer the research question.

### Conclusions and recommendations

Results from the quantitative evaluation showed that both adolescents and parents were positive about the skills training and the focus on emotion and ER skills. Including ER as a transdiagnostic construct in the treatment of adolescents in a CAP outpatient setting was perceived by participants as meaningful. It is thus both feasible and appreciated to teach ER skills in a group format and especially jointly with adolescents and parents together.

Based on participants’ evaluation of the methodological aspects, several clinical and therapeutic recommendations were generated. Despite having several advantages, the group setting itself also had some limitations, especially when group constellations were very heterogeneous. One recommendation is therefore to increase homogeneity in groups when this is feasible in clinical practice. Also, one size doesn’t fit all and complementing the group skills training with individual sessions for a more tailored approach could be beneficial for some participants. Despite some initial hesitation from participants, the big picture tends to fall into place toward the second or third session. Skills trainers thus need to trust the process. A therapeutic skills trainer approach characterized by self-disclosure and an easy-going manner was especially appreciated by participants, and such an approach therefore needs to be practiced. When delivering interventions in a group format to adolescents within a CAP setting, the pedagogy needs to take into account some adolescents’ impaired attention span and difficulty concentrating, and special adjustments need to be made. It is also important to consider if the timing is right for participating in a group ER skills training, based on participants’ symptom severity and level of functioning, in relation to other possible interventions. To optimize generalization of skills, skills trainers need to use several different techniques to encourage adherence to homework assignments and emphasize the importance of generalizing skills.

## Data Availability

The datasets presented in this article are not publicly available to protect participant confidentiality and privacy. Access to the data is subject to ethical permission and participant consent. Requests to access the datasets should be directed to KH, kristina.holmqvist.larsson@liu.se.
